# The Influence of Biological Measures on Strawberry Plant Growth, Yield, and Fruit Quality

**DOI:** 10.3390/plants15060929

**Published:** 2026-03-18

**Authors:** Neringa Rasiukevičiūtė, Armina Morkeliūnė, Ingrida Mažeikienė, Daiga Birzleja, Juozas Lanauskas, Alma Valiuškaitė

**Affiliations:** 1 Laboratory of Plant Protection, Institute of Horticulture, Lithuanian Research Centre for Agriculture and Forestry, Kaunas District, LT-54333 Babtai, Lithuania; armina.morkeliune@lammc.lt (A.M.); daiga.birzleja@lammc.lt (D.B.); alma.valiuskaite@lammc.lt (A.V.); 2 Department of Orchard Plant Genetics and Biotechnology, Institute of Horticulture, Lithuanian Research Centre for Agriculture and Forestry, Kaunas District, LT-54333 Babtai, Lithuania; ingrida.mazeikiene@lammc.lt (I.M.); juozas.lanauskas@lammc.lt (J.L.)

**Keywords:** thyme, biofungicide, bacteria, severity

## Abstract

Finding safe and efficient plant protection measures is one of the major challenges in horticulture. This study evaluated the biological effects of the *Thymus vulgaris* essential oil and of *Bacillus halotolerans* and *B. velezensis* bacterial mixture on strawberry growth and fruit quality properties, as well as on *Botrytis cinerea* severity. The experiment was conducted in a high-tunnel greenhouse with the strawberry cv. Sonsation. Treatments: (1) Control—untreated; (2) Bacteria—with *Bacillus halotolerans* and *B. velezensis*, four times during flowering; (3) Thyme I—*T. vulgaris* essential oil (EO), four times during flowering; (4) Thyme II—*T. vulgaris* EO, four applications supplemented by three additional applications during fruit ripening; (5) Biofungicide I—*Bacillus subtilis* QST 713, four times supplemented by three applications; (6) Biofungicide II—*Clonostachys rosea* J1446, four times during flowering. In the first year (2023), the highest total yield was observed in Thyme II, and in the second year (2024), the highest total yield was observed in Thyme I. The results did not reveal any visual phytotoxic effect on plant leaves. The average fruit diameter increased from 28 mm up to 31 mm in 2023 and from 35 mm to 39 mm in 2024. The average soluble solids content increased from 9.4 to 11.4 °Brix in 2023 and from 7.2 to 7.7 °Brix in 2024. The highest ascorbic acid content in 2023 was observed in Biofungicide II and Biofungicide I treatments, respectively, 79.9 mg % and 75.4 mg %. Similarly, in 2024, the highest ascorbic acid content was observed in Bacteria, Biofungicide I, and Biofungicide II treatments—39.3–40.2 mg %. In vitro, the lowest *B. cinerea* severity on strawberry leaves in 2023 was recorded in Thyme I and Thyme II treatments (~6–7%), while in 2024, the severity in these treatments was higher −20–22%. Thyme treatment showed a stable reduction in *B. cinerea* on leaves in vitro over both years.

## 1. Introduction

### 1.1. Economic Importance and Nutritional Value of Strawberry

Strawberry (*Fragaria* × *ananassa* Duch.) is one of the most widely cultivated fruits worldwide due to its high economic value and desirable organoleptic and nutritional properties [1]. The demand for red fruits continues to increase as consumer awareness of the health benefits associated with strawberry consumption grows [2]. Strawberries are recognized as an important source of bioactive compounds, particularly vitamin C and phenolic constituents, which contribute to their antioxidant capacity and nutritional value [1,3]. Fruit size, yield, and quality are key parameters determining consumer acceptance and economic return in strawberry production systems [4].

### 1.2. The Challenge of Botrytis cinerea and Limitations of Conventional Fungicides

Strawberry production is highly susceptible to various biotic stresses, particularly fungal diseases such as gray mold caused by *Botrytis cinerea*, which can result in substantial yield losses under favorable environmental conditions for pathogen development [5]. Gray mold affects both pre- and post-harvest fruit quality, leading to fruit decay, reduced marketability, and considerable economic losses [6]. The pathogen overwinters on plant tissues and remains quiescent until environmental conditions favor sporulation and infection [7]. To manage fungal diseases, conventional production systems predominantly rely on synthetic fungicides, including single-site fungicides such as methyl benzimidazole carbamates [7]. Although these fungicides can be effective, their repeated use has raised concerns regarding environmental safety, human health risks, and the development of pathogen resistance [8,9]. Intensive pesticide applications may also lead to the accumulation of pesticide residues in fresh fruits and processed products, posing potential acute and chronic health risks to consumers and raising concerns about food safety and environmental sustainability [10,11]. These limitations highlight the need for alternative and more sustainable plant protection strategies.

### 1.3. Biological Control Alternatives and Their Mechanisms

Biological control strategies based on microbial antagonists and plant-derived compounds are increasingly considered as promising alternatives to synthetic fungicides. Beneficial bacteria, particularly *Bacillus* spp., can suppress pathogen development through several mechanisms, including competition for nutrients and space, secretion of antifungal metabolites, and induction of systemic resistance in host plants [12,13]. Previous studies have demonstrated that *Bacillus*-based preparations can reduce *B. cinerea* incidence in strawberries under greenhouse and field conditions [14,15]. Moreover, microbial inoculants have been reported to improve strawberry fruit quality, post-harvest storage life, and overall productivity [16,17,18,19]. Essential oils (EOs) extracted from aromatic plants also exhibit strong antifungal properties. For example, thyme (*Thymus vulgaris*) essential oil contains biologically active terpenoids and phenolic compounds that can inhibit spore germination and mycelial growth of *B. cinerea* [20,21]. In addition, essential oils may act as natural elicitors of plant defense responses, stimulating the production of secondary metabolites and enhancing the resistance to pathogen infection [22]. *T. vulgaris* and other aromatic EOs have been evaluated not only for disease suppression in fruits post-harvest but also for potential integration in pre-harvest applications [22,23]. However, the field performance of essential oils often depends on formulation, concentration, and application timing [24]. Biological measures may also influence plant growth and productivity through multiple mechanisms, including improved nutrient availability in the rhizosphere, phytohormone production, enhanced root development, and activation of plant defense pathways [25,26,27]. The assessment of antioxidant responses in strawberry leaves and fruits following application of different plant protection products can provide insights into the physiological status and stress tolerance of the plants [28,29,30,31,32]. EO is usually used for disease control, but strawberries applied with *Aloysia citrodora* EO had higher enzyme activity, biochemical contents and antioxidant properties. *Aloysia citrodora* EO increased TPC content [33]. These processes may contribute not only to disease suppression but also to improvements in fruit quality parameters such as antioxidant capacity and phenolic content.

### 1.4. Rationale and Objectives of the Study

Despite the growing interest in biological plant protection strategies, the effectiveness of these approaches may vary depending on environmental conditions, treatment combinations, and strawberry cultivars. In particular, long-term or multi-year evaluations of biological control measures remain limited, especially for recently introduced cultivars such as ‘Sonsation’. Understanding the cumulative effects of repeated applications across multiple growing seasons is essential for developing reliable integrated disease management strategies. Therefore, the present 2-year study aimed to evaluate the effects of biological measures, including *Thymus vulgaris* essential oils, *Bacillus halotolerans*, and *Bacillus velezensis*, on strawberry vegetative growth, yield, fruit quality parameters (fruit size, weight, soluble solids content, vitamin C, firmness, and antioxidant properties), and the severity of the *Botrytis cinerea* infection.

## 2. Results

**Figure 1:**
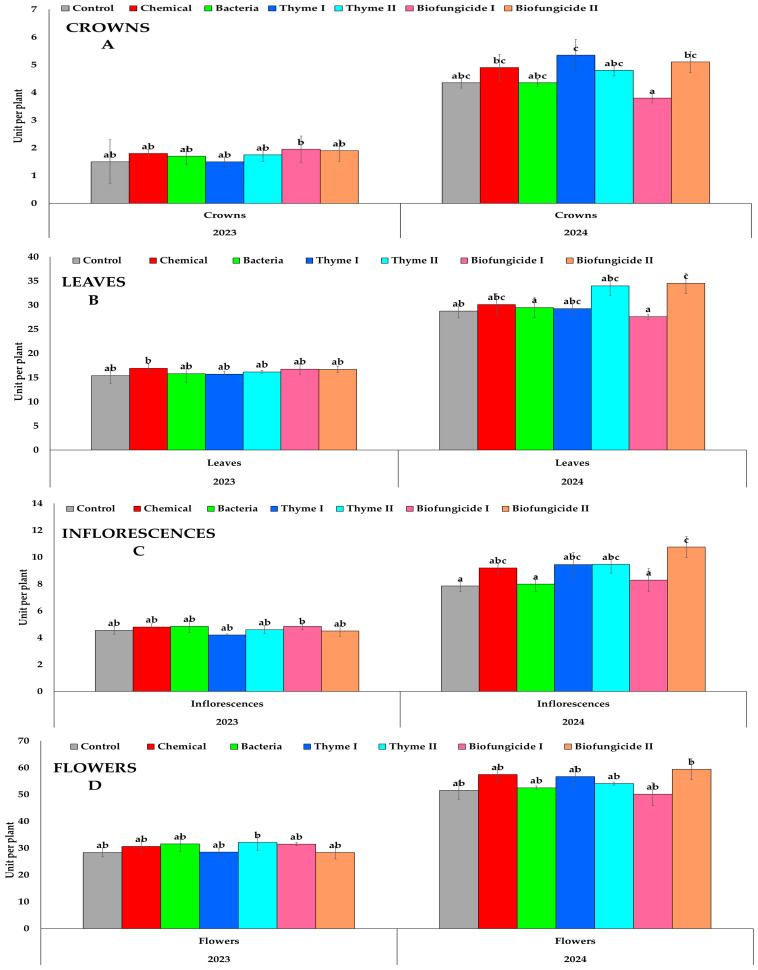
Strawberry cv. Sonsation morphological characteristics development per plant. (A) Crowns, (**B**) Leaves, (**C**) Inflorescences and (**D**) Flowers. Note: the same letter indicates no significant differences between treatments; Duncan’s test (*p* < 0.05). Vertical bars in the figure indicate standard error.

The effect of different biological measures on strawberry morphological characteristics, such as the number of inflorescences, flowers, crowns, and leaves, is provided in Figure 1. The number of crowns per plant was affected by the treatments applied and varied from year to year (Figure 1A). In 2023, the number of crowns per plant changed only a little across all treatments. There were no statistically significant differences among the tested measures. All treatments were classified into the same statistical group, indicating comparable effects on crown formation during the first year of the experiment. However, in 2024, there were significant differences in crown numbers between the treatments. The biggest number of crowns per plant was established in plants treated with Biofungicide II, which exhibited significant differences from other treatments. Plants treated with the Thyme II, Bacteria, and Chemical treatment showed better crown formation, but these treatments were put into overlapping statistical groups. The Control treatment had a moderate number of crowns and did not always show big differences from the other treatments. The Thyme I treatment had fewer crowns than the others, indicating that it had a lower effect on crown development in 2024. In 2024, most treatments had more crowns per plant than in 2023. The treatment effects were not significant in the first year; however, the differences became more pronounced in the second year of the study, suggesting a cumulative or delayed response of strawberry plants to the applied plant protection measures.

The number of leaves per plant differed among treatments and between the two experimental years (Figure 1B). Leaf number per plant showed only minor variations among treatments in 2023, and no significant differences were detected. In contrast, the number of leaves per plant was strongly influenced by all biological measures in 2024. The highest leaf number was recorded in Biofungicide II, which formed a distinct statistical value and exceeded most other treatments. A similarly high leaf number was observed in plants treated with Thyme I and Thyme II. The Bacteria and Chemical treatments showed intermediate leaf numbers that were significantly higher than those of the Control but lower than the most effective treatments. Control resulted in a lower number of leaves compared with some treatments, indicating reduced vegetative growth under untreated conditions. Across all treatments, leaf number per plant was substantially higher in 2024 than in 2023. Crown number showed limited treatment effects in the first year, whereas leaf development responded more clearly to treatment applications in the second year.

The number of inflorescences per plant showed negligible differences among treatments (Figure 1C). No significant differences were observed between the Control and most treatments, as most fell within the same statistical category. The plants treated with Biofungicide I showed a slightly higher number of inflorescences than other treatments; however, this difference was not consistently significant across all comparisons. The development of inflorescences in 2023 was largely consistent across all treatments. In 2024, significant differences in the number of inflorescences per plant were seen among the treatments. Biofungicide II determined the highest number of inflorescences, categorizing them as a distinct statistical group, surpassing all other treatments. The Thyme II, Thyme I, and Chemical treatments exhibited an enhancement in inflorescence production; nevertheless, these treatments fell within overlapping statistical groups and displayed intermediate values. The Bacteria, Biofungicide I, and Biofungicide II treatments produced a considerable quantity of inflorescences. In 2024, the Control treatment produced the fewest inflorescences. Unlike the relatively similar response observed in the first year, inflorescence formation in the second year showed a marked response to the treatment, indicating greater differentiation among biocontrol measures over time.

The number of flowers per plant (Figure 1D) showed relatively small variation among treatments in 2023. No significant differences were detected between the control and the majority of applied treatments, as all treatments that were assigned to the same or overlapping statistical groups. The plants treated with Thyme II exhibited a slightly higher mean number of flowers compared with the Control treatment and several other treatments. However, this increase was not significant. The Chemical, Bacteria, Thyme I, Biofungicide I, and Biofungicide II treatments resulted in comparable flower numbers, indicating that none of the applied plant protection strategies had a distinct effect on flower formation during the first year of the experiment. Flowers per plant increased across all treatments in 2024 compared with 2023. Although numerical differences among treatments were observed, a statistical analysis revealed limited differentiation, with most treatments belonging to the same statistical group. The highest mean value of flowers was recorded in plants treated with Biofungicide II, followed by the Chemical and Thyme I treatments. However, these values did not differ significantly from the Control or other treatments. The plants treated with Bacteria and Biofungicide I treatments produced a moderate number of flowers, which remained statistically comparable to the other treatments. Overall, the flower production in 2024 was relatively uniform across treatments despite higher absolute values. In general, the number of flowers per plant was higher in 2024 than in 2023, indicating enhanced morphological characteristic development in the second year of the experiment. Besides both years, no visible phytotoxic effect symptoms were observed on the strawberry plant leaves due to the treatments, including essential oils.

**Figure 2:**
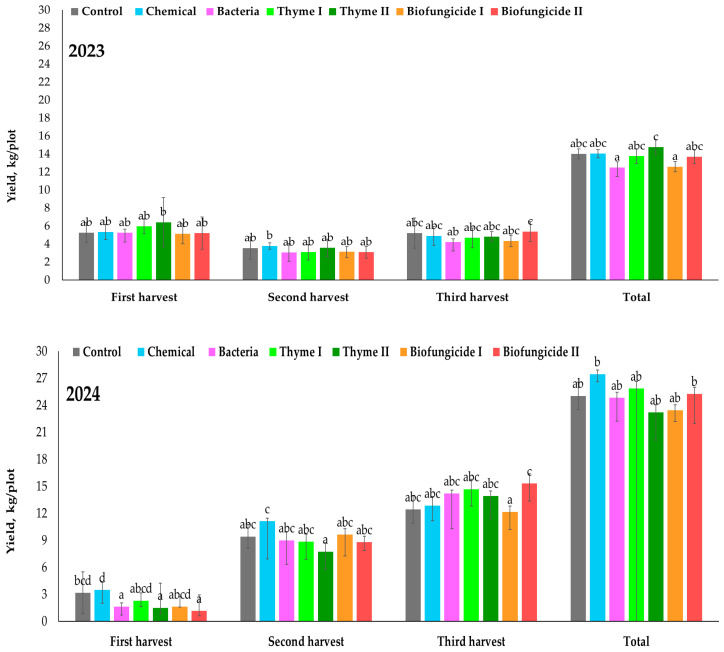
First, second, and third harvest and total yield of strawberry cv. Sonsation. Note: the same letter indicates no significant differences between treatments; Duncan’s test (*p* < 0.05). Vertical bars in the figure indicate standard error.

The yield varied between harvests, but different biocontrol measures had a very limited effect on the yield in 2023 (Figure 2).

No statistically significant difference was observed between the Control and most treatments, as the majority of treatments (the Chemical, Bacteria, Thyme I, Biofungicide I, and Biofungicide II treatments) were assigned to the same statistical group. The Thyme II (6.42 kg) treatment yield was slightly higher compared with the Control (5.26 kg) and other treatments. However, the Chemical (5.34 kg), Bacteria (5.24 kg), Thyme I (5.97 kg), and Biofungicide I-II (5.13–5.21 kg) treatments did not differ significantly from the Control treatment. In the second harvest, yields were generally lower compared with the first harvest across all treatments. The Chemical treatment (3.80 kg) showed a slightly higher yield. Greater variability among treatments was observed during the third harvest. The Biofungicide II (5.40 kg) treatment produced the highest yield during the third harvest, indicating a significantly higher yield compared with several other treatments. In contrast, the Bacteria (4205 g) and Biofungicide I (4.33 kg) treatments yielded less than the others. The Control, Chemical, Thyme I, and Thyme II treatments showed an intermediate yield. The total yield per plot, calculated as the sum of all harvests, differed significantly among treatments. The highest total yield was recorded for the Thyme II (14.78 kg) treatment. The Bacteria (12.51 kg) and Biofungicide I (12.59 kg) treatments had the lowest total yield. The Chemical (14.04 kg) and Control (14.01 kg) treatments had an intermediate total yield. The Thyme I (13.76 kg) and Biofungicide II (13.69 kg) treatments showed a moderate yield. Strawberry yield per plot in 2024 varied across harvest times and treatments (Figure 2). The treatment effects differed across harvest times, and significant differences were shown in the total yield. During the first harvest, the Chemical treatment had the highest yield (3.48 kg). The Thyme I (2.30 kg) and Biofungicide I (1.63 kg) treatments were intermediate. In the second harvest, the yield increased compared with the first harvest. The Chemical treatment (9.44 kg) showed the highest yield. The Thyme II treatment (7.76 kg) had a significantly lower yield at the second harvest compared with the Chemical treatment. The third harvest showed the clearest differentiation among treatments. The Biofungicide II (15.32 kg) treatment had the highest yield, but Biofungicide I (12.19 kg) treatment had the lowest yield during the third harvest. Total yield per plot differed significantly among treatments. The Chemical (27.46 kg) and Thyme I (25.85 kg) treatments had the highest total yield. The Biofungicide II (25.27 kg) and Bacteria (24.83 kg) treatments were in the middle of the total yield. All the remaining treatments, including the Control treatment, were not significantly different from one another and were not consistently different from the highest-yielding group. When comparing the two years, the total yield was generally higher and more evenly distributed among treatments in 2024. While the Thyme II treatment performed well in 2023, the relative advantage was lower in 2024. However, the Thyme I treatment acted better in 2024 than in 2023. In contrast, the Biofungicide II treatment maintained high productivity across both years, showing stable performance in different years.

**Figure 3:**
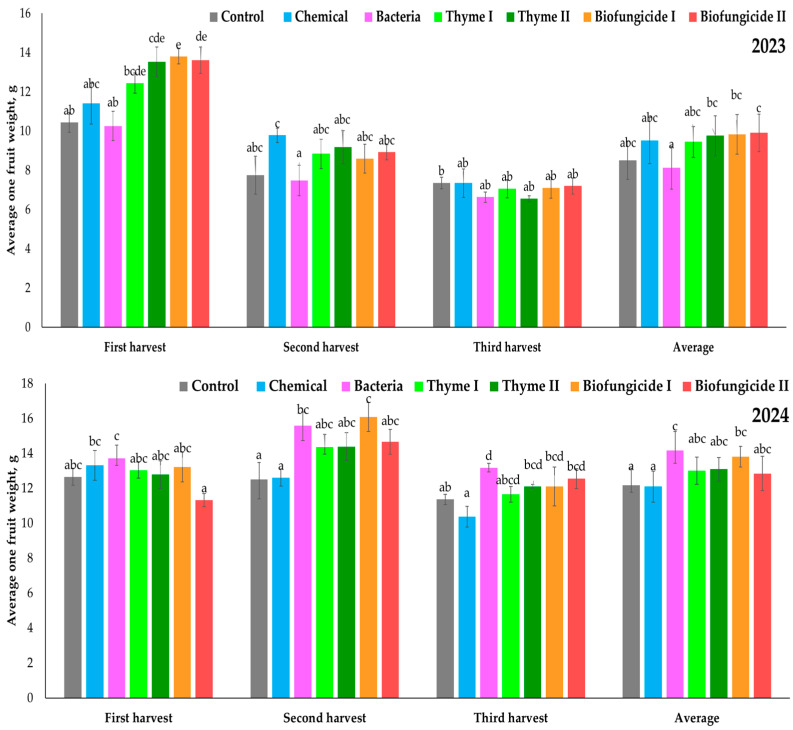
Strawberry cv. Sonsation average single fruit weight, g. Note: the same letter indicates no significant differences between treatments; Duncan’s test (*p* < 0.05). Vertical bars in the figure indicate standard error.

The average fruit weight of strawberries in 2023 was significantly affected by the treatment and harvesting time (Figure 3). During the first harvest, the average fruit weight varied significantly among treatments. The lowest fruit weight was in the Control (10.44 g) and Bacteria (10.26 g) treatments, which did not differ significantly from each other. The Chemical treatment (11.40 g) resulted in a slightly higher fruit weight. Both the Thyme I (12.44 g) and Thyme II (13.54 g) treatment had significantly heavier fruits. The highest average fruit weights were observed in the Biofungicide I (13.8 g) and Biofungicide II (13.62 g) treatments, indicating significantly higher fruit weight compared with the Control (10.44 g) and Bacteria (10.26 g) treatments. During the second harvest, the average fruit weight decreased in all treatments compared with the first harvest. Significant differences among treatments remained. The Chemical treatment (9.78 g) had the highest average fruit weight, significantly higher than that of the Control (7.76 g) and Bacteria (7.49 g) treatments. During the third harvest, the average fruit weight further decreased, and the differences among treatments were lower. The Bacteria (6.64 g) and Thyme II (6.55 g) treatments had the lowest fruit weights. The average fruit weight differed significantly across treatments. The Control (8.52 g) and Bacteria (8.13 g) treatments had the lowest average fruit weights. The Chemical treatment (9.51 g) had a moderate increase but did not differ significantly from the Control treatment. In contrast, the Thyme I (9.45 g), Thyme II (9.76 g), Biofungicide I (9.84 g), and Biofungicide II (9.91 g) treatments resulted in higher average fruit weights.

During the first harvest in 2024, significant differences in average fruit weight were observed among treatments. The Bacteria (13.72 g) and Chemical (13.31 g) treatments had the highest fruit weight, indicating significantly heavier fruits compared with other treatments. The Biofungicide II (11.32 g) treatment had the lowest average fruit weight, significantly different from the Bacteria and Chemical treatments. During the second harvest, the treatment effects were more pronounced. The highest average fruit weight was recorded for the Biofungicide I (16.09 g) treatment, which differed significantly from the Control (12.51 g) and Chemical (12.61 g) treatments. The Bacteria (15.60 g) treatment resulted in high fruit weight. In Thyme I (14.35 g), Thyme II (14.39 g), and Biofungicide II (14.66 g) treatments, fruits were of intermediate weight. The Control and Chemical treatments had the lowest fruit weights. During the third harvest, the Bacteria (13.17 g) and Biofungicide II (12.57 g) treatments resulted in significantly heavier fruits in comparison with the other treatments. The Chemical (10.37 g) and Control (11.37 g) treatments had the lowest fruit weight. The average data showed that the highest fruit weight in 2024 was observed in the Bacteria (14.17 g) and Biofungicide I (13.80 g) treatments.

**Figure 4:**
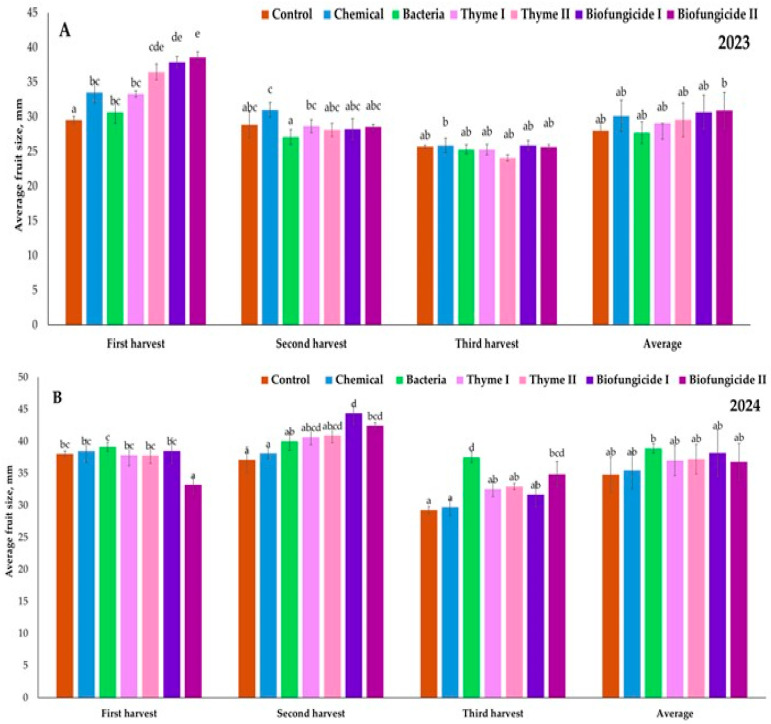
Strawberry cv. Sonsation average fruit diameter, mm. (**A**) year 2023, (**B**) year 2024. Note: The same letter indicates no significant differences between treatments; Duncan’s test (*p* < 0.05). Vertical bars in the figure indicate standard error.

In 2023, during the first harvest, the average fruit diameter varied from 29.50 mm in the Control treatment up to 38.58 mm in the Biofungicide II treatment (Figure 4A). The fruits that were closest to the largest were also in the Biofungicide I (37.85 mm) and Thyme II (36.45 mm) treatments. The smallest fruits were observed in the Control (29.50 mm) and Bacteria (30.65 mm) treatments. During the second harvest, the fruit size was smaller across all treatments, ranging from 27.08 mm (in the Bacteria treatment) to 30.98 mm (in the Chemical treatment). Most treatments exhibited similar fruit sizes, with a diameter of around 28–29 mm. During the third harvest, the fruit size was varying from 24.05 mm (in the Thyme II treatment) to 25.85 mm (in the Chemical and Biofungicide I treatments). The differences among treatments were quite minor at the third harvest. The average data in 2023 showed that the highest average fruit size was observed in the Biofungicide II (30.90 mm) and Biofungicide I (30.63 mm) treatments, with the Chemical treatment following at 30.10 mm. The average fruit size was smallest in the Bacteria (27.68 mm) treatment, closely followed by the Control treatment at 28.00 mm. In 2024, the average fruit size significantly increased compared to 2023 across all treatments and harvest times. During the first harvest, the fruit diameter varied from 33.18 mm (in the Biofungicide II treatment) to 39.15 mm (in the Bacteria treatment) (Figure 4B). In most treatments, fruit diameter was over 37 mm, with the Chemical treatment being 38.50 mm and the Biofungicide I treatment being 38.48 mm. During the second harvest, the fruit size reached its maximum for all treatments. The biggest fruit diameter was found in the Biofungicide I treatment (44.35 mm), followed by the Biofungicide II treatment (42.40 mm) and the Thyme II treatment (40.88 mm). The Bacteria treatment produced fruits of 40.00 mm diameter, while the Control and Chemical treatments had fruits of smaller diameter—37.08 mm and 38.10 mm, respectively. During the third harvest, the fruit diameter varied from 29.25 mm in the Control treatment to 37.53 mm in the Bacteria treatment. The Biofungicide II treatment also produced quite large fruits (34.83 mm). The average data for 2024 showed that the largest average fruit diameter—38.89 mm—was determined by the Bacteria treatment, followed by the Biofungicide I treatment—38.16 mm—and the Thyme II treatment —37.19 mm. The Control treatment had the smallest average fruit diameter—34.78 mm. In all treatments, the average fruit size in 2024 was significantly larger than in 2023. The average fruit size increased from 28 to 31 mm in 2023 to 35–39 mm in 2024.

**Figure 5:**
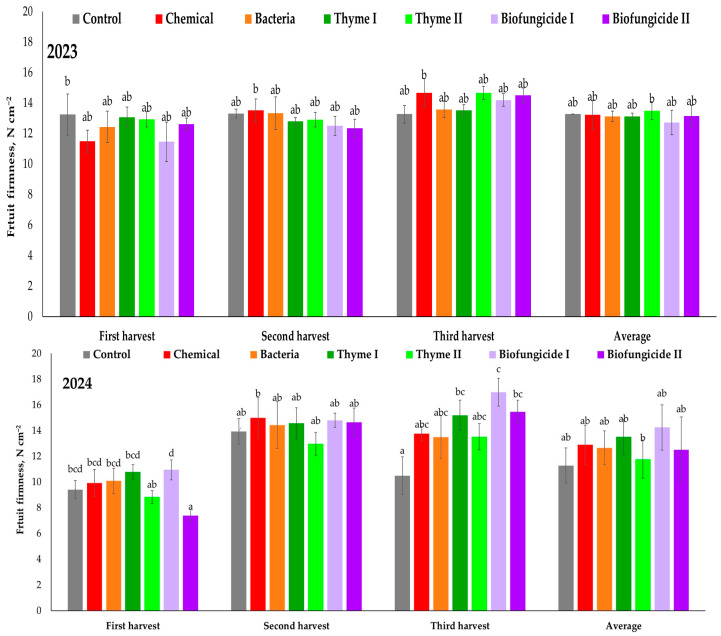
Strawberry cv. Sonsation fruit firmness, N cm^−2^. Note: the same letter indicates no significant differences between treatments; Duncan’s test (*p* < 0.05). Vertical bars in the figure indicate standard error.

In 2023, during the first harvest, fruit firmness varied from 11.48 N cm^−2^ in the Chemical treatment to 13.24 N cm^−2^ in the Control (Figure 5) treatment. The high fruit firmness was also observed in the Thyme I (13.07 N cm^−2^) and Thyme II (12.92 N cm^−2^) treatments. The Biofungicide I (11.46 N cm^−2^) and Chemical (11.48 N cm^−2^) treatments had a negative effect on fruit firmness during the first harvest. No significant changes were detected across the treatments. During the second harvest, the firmest fruits were found in the Chemical (13.51 N cm^−2^), Bacteria (13.33 N cm^−2^), and Control (13.29 N cm^−2^) treatments. The Biofungicide I (12.50 N cm^−2^) and Biofungicide II (12.35 N cm^−2^) treatments showed the lowest fruit firmness. During the third harvest, fruit firmness varied from 13.27 N cm^−2^ (in the Control treatment) to 14.67 N cm^−2^ (in the Chemical treatment). However, during the third harvest, the firmest fruits were observed in the Thyme II (14.66 N cm^−2^), Chemical (14.67 N cm^−2^), and Biofungicide II (14.49 N cm^−2^) treatments. In 2023, the average highest fruit firmness was observed in the Thyme II treatment (13.49 N cm^−2^) and the lowest in the Biofungicide II (12.71 N cm^−2^) treatment. In 2024, fruit firmness showed increased variability among treatments, especially during the third harvest and in the overall average (Figure 5). During the first harvest, firmness varied from 7.38 N cm^−2^ in the Biofungicide I treatment to 10.94 N cm^−2^ in the Thyme I treatment. The firm fruits were also found in the Thyme I treatment—10.78 N cm^−2^. During the second harvest, fruit firmness significantly increased across all treatments. The firmest fruits were observed in the Biofungicide II (14.65 N cm^−2^) and Chemical (14.99 N cm^−2^) treatments, 14.65 N cm^−2^ and 14.99 N cm^−2^, respectively. During the third harvest, bigger differences in fruit flesh firmness between treatments were established. The firmest fruits were after the Biofungicide I and Biofungicide II treatments, 16.98 N cm^−2^ and 15.46 N cm^−2^, respectively. The softest fruits were found in the Control treatment—10.49 N cm^−2^. Overall, in 2024, the firmest fruits were observed after the Biofungicide II (14.24 N cm^−2^) and Thyme I (13.51 N cm^−2^) applications, whereas the lowest average fruit firmness was found in the Control treatment—11.28 N cm^−2^. In 2023, fruit firmness was more consistent throughout all harvests and treatments, while in 2024, it exhibited greater variability among treatments, especially within the second and third harvests.

**Figure 6:**
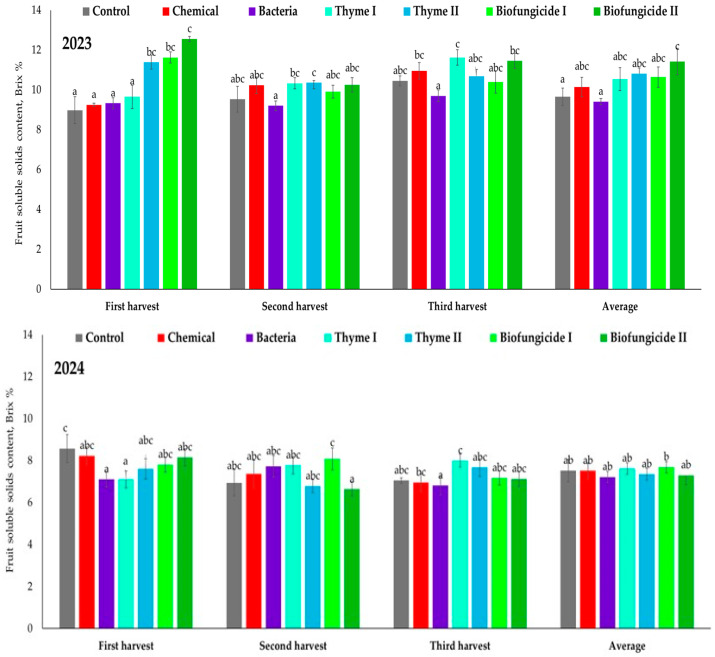
Strawberry cv. Sonsation fruit soluble solids content (Brix %) Note: the same letter indicates no significant differences between treatments; Duncan’s test (*p* < 0.05). Vertical bars in the figure indicate standard error.

The strawberry fruit’s soluble solids content (SSC) ranged from 8.98 to 12.55 °Brix depending on treatment and harvest time in 2023 (Figure 6). During the first harvest, the SSC ranged from 8.98 °Brix in the Control treatment to 12.55 °Brix in the Biofungicide II treatment. The highest SSC was found in the Biofungicide I (11.63 °Brix) and Thyme II (11.38 °Brix) treatments, while the Chemical (9.25 °Brix) and Bacteria (9.33 °Brix) treatments had lower SSC compared with the Control treatment. During the second harvest, the SSC was more uniform, ranging from 9.20 °Brix (in the Bacteria treatment) to 10.35 °Brix (in the Thyme II treatment). The SSC in the Control and Chemical treatments was 9.53 °Brix and 10.23 °Brix, respectively. The SSC increased in several treatments, ranging from 9.70 °Brix (in the Bacteria treatment) to 11.63 °Brix (in the Thyme I treatment) during the third harvest. Relatively high SSC values were also observed after the Chemical (10.95 °Brix) and Biofungicide II (11.45 °Brix) treatments. The average SSC in 2023 ranged from 9.41 °Brix (in the Bacteria treatment) to 11.42 °Brix (in the Biofungicide II treatment). In 2024, the SSC was generally lower than in 2023 and varied within a narrower range between treatments. During the first harvest, the SSC ranged from 7.38 °Brix (in the Biofungicide II treatment) to 8.57 °Brix (in the Control treatment). The SSC in the Bacteria treatment was 7.11 °Brix, while in the Thyme II treatment, it was 7.60 °Brix. During the second harvest, the SSC varied between 6.63 °Brix (in the Biofungicide II treatment) and 8.01 °Brix (in the Biofungicide I treatment). In the Control and Chemical treatments, the SSC was 6.94 °Brix and 7.36 °Brix, respectively. During the third harvest, the SSC ranged from 6.82 °Brix (in the Bacteria treatment) to 8.00 °Brix (in the Thyme I treatment). The average SSC in 2024 ranged from 7.22 °Brix (in the Chemical treatment) to 7.68 °Brix (in the Thyme II treatment). Across all treatments, the SSC was consistently higher in 2023 than in 2024. The average SSC in 2023 was within the range of 9.4–11.4 °Brix and 7.2–7.7 °Brix in 2024, indicating a substantial year effect on sugar accumulation in strawberry fruits.

To estimate specific changes in the nutritional quality of strawberries, we evaluated the antioxidant system response, including total phenolic compounds (TPCs), FRAP, ABTS, DPPH, and ascorbic acid content (Table 1). Concerning ascorbic acid content, there was a slight variation between treatments in both experimental years. In 2023, ascorbic acid content ranged from 51.0% in the Controlto 79.9 mg % in the Biofungicide II. To estimate specific changes in the nutritional quality of strawberries, we evaluated the antioxidant system response, including total phenolic compounds (TPCs), FRAP, ABTS, DPPH, and ascorbic acid content. The highest ascorbic acid content was also in the Biofungicide I treatment—75.4 mg %. The Thyme I (60.4 mg %), Bacteria (59.3 mg %), and Thyme II (58.5 mg %) treatments had no effect on ascorbic acid content changes. In 2024, similar tendencies were observed: the highest ascorbic acid content was established in the Biofungicide I (40.2 mg %), Biofungicide II (39.9 mg %), and Bacteria (39.3 mg %) treatments.

In 2023, the TPCs ranged from 66.9 mg g^−1^ SM (in the Control treatments) up to 86.0 mg g^−1^ SM (in the Biofungicide I and II treatments). The ABTS radical scavenging activity varied between 634.0 mM TE g^−1^ SM (Thyme II) and 778.3 mM TE g^−1^ SM (Thyme I). FRAP ranged from 1466.7 µmol g^−1^ SM (Thyme II) to 1603.1 µmol g^−1^ SM (Biofungicide I).

In 2024, antioxidant activity values were generally higher than in 2023, particularly for DPPH, ABTS, and FRAP assays. The DPPH activity ranged from 934.7 mM TE g^−1^ SM (the Chemical treatment) to 1344.4 mM TE g^−1^ SM (Thyme I). In 2024, the highest TPCs were found in the Bacteria (89.3 mg g^−1^ SM) and Chemical (86.2 mg g^−1^ SM) treatments. The lowest TPC content was found in the Thyme I treatment—72.3 mg g^−1^ SM. The application of the Thyme I treatment in both 2023 and 2024 resulted in the higher antiradical DPPH, 939.5 mM TE g^−1^ SM and 1344.4 mM TE g^−1^ SM, respectively. The DDPH ranged from 864.3 mM TE g^−1^ SM (Control) up to 939.5 mM TE g^−1^ SM (Thyme I) in 2023. But in 2024, the DPPH ranged from 934.7 mM TE g^−1^ SM (Chemical) to 1344.4 mM TE g^−1^ SM (Thyme I). The differences in phenolic compound content significantly influence the antioxidant activity. The decrease in phenolic content could be an indication that treatments are working well. The Thyme I treatment showed a similar increase in ABTS antiradical activity in both years. However, the Biofungicide II treatment resulted in a decrease in the ABTS antiradical activity. In 2023, the highest FRAP antioxidant activity was found in the Biofungicide I (1603.1 µmol g^−1^ SM) treatment, followed by the Thyme I (1597.5 µmol g^−1^ SM) treatment. In 2024, FRAP was highest in the Chemical (2035.2 µmol g^−1^ SM) treatment, followed by the Thyme I (1997.7 µmol g^−1^ SM) and Bacteria (1990.1 µmol g^−1^ SM) treatments. The ABTS values ranged from 939.5 mM TE g^−1^ SM (Control) to 1121.8 mM TE g^−1^ SM (Chemical). The Biofungicide I treatment also resulted in high ABTS activity (1088.5 mM TE g^−1^ SM). High FRAP values were also recorded for the Thyme I (1997.7 µmol g^−1^ SM) and Bacteria (1990.1 µmol g^−1^ SM) treatments. Overall, the antioxidant system response (DPPH, ABTS, and FRAP) was generally higher in 2024 than in 2023, whereas ascorbic acid content was higher in 2023 across most treatments. The biofungicide and thyme-based treatments frequently enhanced antioxidant system response compared with the Control treatment, although responses differed depending on the assay and year.

**Figure 7:**
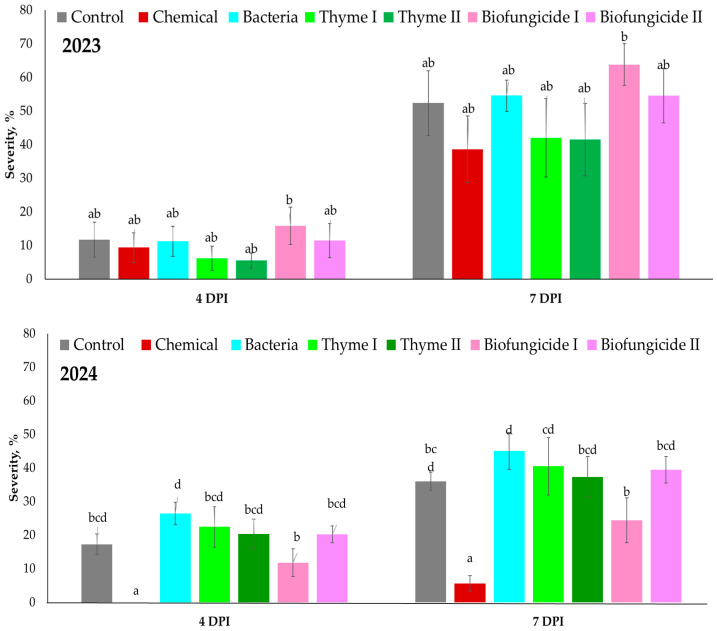
The *B. cinerea* severity (%) on the strawberry cv. Sonsation leaves. Note: DPI—days post inoculation. The same letter indicates no significant differences between treatments; Duncan’s test (*p* < 0.05). Vertical bars in the figure indicate standard error.

The in vitro experiment revealed that the *B. cinerea* severity on strawberry leaves varied between days after inoculation (Figure 7). In 2023, four days after inoculation (4 DPI), the *B. cinerea* severity rate of gray mold was fairly low across all treatments, ranging from about 5% to 18%. The severity in the Control treatment was 12%, whereas in the Chemical treatment, the severity was 10%. The Bacteria treatment results were similar, at approximately 12%. The Thyme I (~7%) and Thyme II (~6%) treatments had lower severity, while the Biofungicide I treatment had the highest severity (17–18%). The Biofungicide II treatment had a medium severity 12% at 4 DPI. At 7 DPI, the severity increased in all treatments. The severity in the Control treatment was around 55%, while in the Chemical treatment, it was about 40%. The severity in the Bacteria treatment was 58–60%. The Thyme I and Thyme II treatments had about 43–45% disease severity. The Biofungicide I treatment, on the other hand, had the highest severity of gray mold at this point (around 70%), whereas the Biofungicide II treatment had about 55–58%. In 2024, severity was higher at 4 DPI; it had more distinct treatment-related variations. At 4 DPI, gray mold severity ranged from around 10% to 25%. In the Control treatment, gray mold severity was 15–17%, while in the Chemical treatment, it was 0–2%. At this point, the Bacteria treatment had the highest rate of success (~25%). The Thyme I and Thyme II treatments both caused moderate amounts of gray mold severity, at 20–22%, while the Biofungicide I treatment had the lowest severity, around 12%. The severity of the Biofungicide II treatment was about 20%. At 7 DPI, in the Control treatment, gray mold severity was approximately 35–38%, whereas in the Chemical treatment, it was only 5–7%. However, in the Bacteria treatment, gray mold severity was the highest, about 45–48%. Thyme treatments resulted in intermediate severity, 38–42%. In general, gray mold severity was higher in 2024 than in 2023. Thyme treatments showed stable disease decrease throughout both years, but the Biofungicide treatments exhibited inconsistent performance depending upon the year and assessment period.

## 3. Discussion

The present 2-year study demonstrated that biological plant protection measures had a certain effect on strawberry vegetative growth, yield, fruit quality attributes, antioxidant system response, and *B. cinerea* severity, with year-dependent effects. The variability of the impact measures aligns with previous findings indicating that the performance of biological control agents and plant-derived products is strongly modulated by environmental conditions, crop phenology, and seasonal factors [28,34,35]. Vegetative growth parameters, particularly crown and leaf number, responded more clearly to treatments in the second year of the experiment, suggesting a cumulative or delayed plant response to repeated applications of biological measures. Similar delayed effects of *Bacillus*-based biocontrol agents on strawberry vegetative development have been reported by Tsotetsi et al. [15] and Cawoy et al. [13], who linked enhanced plant growth to improved nutrient availability and induced systemic resistance mechanisms. The higher crown and leaf formation observed under the Biofungicide II treatment and both thyme treatments in 2024 support the hypothesis that microbial antagonists and essential oils may indirectly promote vegetative growth by reducing biotic stress pressure. Yield responses varied markedly between the years. In 2023, the Thyme I treatment resulted in the highest total yield, whereas in 2024, chemical protection and the Biofungicide II treatment were superior. Comparable inconsistencies were observed by Mounaimi et al. [36] and Ezzat et al. [37], who reported that biocontrol-based strategies may outperform chemical control in certain seasons but not consistently across the years. In similar experiments, plants treated with Thymus and Juniperus essential oils showed a final yield increase [28], and *Bacillus velezensis* CE 100 increased strawberry yield compared to the non-inoculated control [38]. The relatively stable performance of the Biofungicide II treatment across both seasons suggests greater robustness of fungal antagonists such as *Clonostachys rosea*, as also reported by Romanazzi et al. [32] in induced resistance studies. Fruit size and weight were significantly enhanced in 2024, particularly under *Bacillus*-based treatments, indicating improved assimilate partitioning and sink strength. Similar increases in fruit size following *Bacillus* applications have been documented by Niu et al. [14] and Battino et al. [39], who associated microbial activity with improved plant physiological status. Biological non-chemical alternatives to chemical fungicides show promising results in tunnel and glasshouse conditions [7]. The possibilities of biological control can be expanded by combining different modes of microorganism actions; for example, *Bacillus* spp. could display multi-faceted biocontrol activity [12].

In contrast, the SSC was consistently higher in 2023, highlighting the strong influence of weather conditions on sugar accumulation, as previously emphasized by Kadir et al. [4]. Fruit flesh firmness differed between years and treatments. The Thyme and Biofungicide I and II treatments maintained or increased firmness compared to the Control treatment, which agrees with the findings of Falleh et al. [21], who reported that thyme essential oils can preserve fruit structural integrity by reducing pathogen-induced softening. Antioxidant activity (DPPH, ABTS, FRAP) was higher in 2024, particularly in thyme-treated plants, whereas ascorbic acid content was higher in 2023.

Essential oils have high antimicrobial activity from their bioactive compounds [22]. This agrees with Huang et al. [20] and Romanazzi et al. [32], who demonstrated that essential oils and resistance inducers can stimulate defense-related metabolic pathways, leading to increased phenolic content and antioxidant capacity in strawberry tissues. In contrast, vitamin C accumulation is more dependent on environmental and ripening conditions [1,40,41,42]. Gray mold severity was generally higher in 2023 than in 2024. Thyme treatments consistently reduced the *B. cinerea* severity compared with the Control treatment, especially at 7 DPI.

These results confirm earlier findings of Ben Jemâa et al. [21] and Bassolé and Juliani [22], who reported strong antifungal activity of thyme essential oil against *B. cinerea.* The moderate but stable performance of the Biofungicide II treatment corresponds with previous studies demonstrating effective suppression of gray mold by *Clonostachys rosea* under controlled and field conditions [43]. Variable efficacy of Bacillus-based treatments observed in this study has also been reported elsewhere and is attributed to strain specificity and environmental compatibility [13]. The enhanced accumulation of phytochemicals in strawberry fruits observed in this study may be associated with several physiological and biochemical processes triggered by biological treatments. Beneficial microorganisms and other biological agents can improve nutrient availability in the rhizosphere, which enhances plant nutrition and stimulates metabolic activity [25]. An improved nutrient status and microbial interactions may also promote the biosynthesis of secondary metabolites, including phenolic compounds, flavonoids, and antioxidant molecules that contribute to fruit quality. Furthermore, plant–microbe interactions are known to activate plant defense mechanisms and induce systemic resistance, which is often accompanied by increased production of phenolic compounds involved in plant protection [27]. In addition, microbial activity may influence phytohormone balance and root development, indirectly supporting the accumulation of bioactive compounds in fruits [26]. These mechanisms together may explain the increased phytochemical content observed in strawberries treated with biological measures. The observed changes in phytochemical composition may also be related to alterations in carbon allocation and plant stress responses. Under the influence of biological treatments, plants may redistribute carbon resources between primary growth and the synthesis of secondary metabolites, which are often associated with fruit quality and antioxidant capacity [25,27]. In addition, moderate biotic or abiotic stress can stimulate the production of secondary metabolites, including phenolic compounds and flavonoids, as part of plant defense mechanisms [44]. Plant–microbe interactions may further regulate these processes through microbial signaling molecules that influence plant metabolic pathways and activate defense-related gene expression [25]. These mechanisms may contribute to enhanced phytochemical accumulation observed in strawberry fruits.

The response to biological treatments may vary among strawberry cultivars due to genetic differences influencing plant physiology, metabolism, and stress tolerance. Different cultivars can exhibit variations in nutrient uptake efficiency, root architecture, and metabolic pathways involved in the synthesis of secondary metabolites. These differences may lead to cultivar-specific effects on plant growth, yield, and phytochemical accumulation in fruits. Similar variability among strawberry cultivars has been reported in previous studies, indicating that genotype plays an important role in determining the effectiveness of biological treatments [1]. The effectiveness of biological measures may also depend strongly on environmental conditions. Factors such as soil properties, temperature, moisture availability, and native microbial communities can influence the survival and activity of beneficial microorganisms and consequently affect their interaction with plants. These environmental variables may lead to variability in plant growth responses, yield formation, and phytochemical accumulation in strawberry fruits. Previous studies have also highlighted that the performance of biological agents can vary significantly under different environmental and soil conditions [25,45].

Although the present study demonstrated the potential of biological measures to influence strawberry vegetative growth, yield formation, fruit quality attributes, antioxidant activity, and gray mold suppression, further research is required to better understand their long-term performance and practical applicability. Future studies should evaluate these treatments under a wider range of climatic and soil conditions to clarify their environmental stability and adaptability across different strawberry production systems. In addition, the optimization of application timing, frequency, and concentration may improve the consistency and effectiveness of biological treatments. Investigating potential synergistic interactions between different biological agents, such as microbial antagonists and plant-derived compounds, could further enhance disease control and plant performance. The economic analyses comparing treatment costs, yield benefits, and quality improvements would also be valuable for assessing their feasibility for commercial production. Such research would contribute to the development of more sustainable and integrated pest management (IPM) strategies in strawberry cultivation.

## 4. Materials and Methods

### 4.1. Experimental Site

The 2-year strawberry cultivar Sonsation experiments were carried out at the Lithuanian Research Centre for Agriculture and Forestry (LAMMC), Institute of Horticulture (IH), in a high-tunnel greenhouse. Sonsation is a productive June-bearing cultivar, well-suited for greenhouse cultivation. The strawberry plants were grown in peat substrate (Terraerden, Rucava, Latvia) with NPK (140–210, 160–240, 180–270 mg L^−1^) and microelements such as Mn, Cu, Mo, B, Zn, Fe; pH_H_2_O_ 5.5–6.5, electrical conductivity (EC) < 1.50 ms cm^−1^. The 15 × 19 cm plastic pots filled with 3 L of substrate were used for each strawberry plant. The plants were watered, maintaining appropriate substrate humidity and fertilizing as needed. The treatments are provided in Table 2, and each treatment had four replicates, 32 plants per replicate. The experiment was set up as a randomized complete block. The applications on the plant started from 10% flowering (BBCH 61–65). The temperature and humidity were recorded with a data logger, Termio+ (Termoprodukt, Bielawa, Poland), and are provided in Table 3.

### 4.2. Essential Oil

The *Thymus vulgaris* essential oil was extracted by hydrodistillation using a Clevenger distillation system (Glassco, Dehli, India). The *T. vulgaris* chemical composition: Thymol 52.22%, p-cymene 12.37%, γ-terpinene 8.39% [46].

### 4.3. Bacteria Isolates

The *Bacillus halotolerans* (two isolates Bil-LT1_1, Bil-LT1_2) and *B. velezensis* (two isolates Cran-LT_1_8, Ling-NOR_4_15) [47] were used as a mixture in equal proportions, resulting in the final ~10^−6^ CFU/L concentration.

### 4.4. Measurements

The experiments were conducted according to the EPPO standards PP1/190(3) and PP1/16(3) [39,43,46]. Plant morphological characteristics, such as inflorescences, crowns, and the number of leaves and flowers, were evaluated as units per plant in May. In each replicate, six plants were selected for morphological measurements [48,49]. The phytotoxicity evaluation was done according to the EPPO standard PP1/135(4) [50].

Strawberries were harvested three times (in 2023 on (a) 5 June, (b) 8 June, (c) 12 June and in 2024 on (a) 29 May, (b) 3 June and (c) 6 June). During pickup, we evaluated yield (g/plot), one-fruit weight (g/fruit), and fruit size (mm/fruit), determined by the maximum diameter of the equatorial section. Randomly selected 10 fruits per replicate were used to determine firmness using the texture analyser, FR-5105 (FR-5105 Lutron Fruit Hardness Tester FR-5120, Lutron Electronic Enterprise Co., Ltd., Taipei, Taiwan). The firmness was measured on two opposite equatorial fruit sides by penetrating a 6 mm diameter flat-head stainless cylindrical probe into fruit flesh. The fruit soluble solids content (Brix, %) was measured with a digital pocket refractometer, Atago PAL-1 (Atago Co., Ltd., Tokyo, Japan). The fruit ascorbic acid (Vitamin C) level was determined trichromatically using a 2,6-dichlorphenolindophenol sodium salt solution (AOAC, 1990) [51].

### 4.5. Biochemical Properties

The leaves for the biochemical properties evaluation were collected after the fourth application (BBCH 71-77). Five leaves per replicate were collected. The extracts for the analysis were prepared from lyophilized strawberry leaves, 0,05 g, diluted with 5 mL 80% methanol for 24 h at 4 °C. The samples were then centrifuged 15 min at 4000× *g*, and the supernatant was separated from the strawberry leaf material. The biochemical properties analysis was performed from the extracts. The total phenolic content (TPC, mg g^−1^ SM), DPPH (2-diphenyl-1-picrylhydrazyl, mM Trolox equivalents (TE) g^−1^ dry mass (SM)) measured at 515 nm, ABTS (2,2′-azino-bis (3-ethylbenzothiazoline-6-sulphonic acid, mM TE g^−1^ SM) measured at 734 nm, and Fe^2+^ reducing antioxidant power assay (FRAP, µmol g^−1^ SM) were measured at 593 nm, determined as described by Laužikė et al. [52] spectrophotometrically (M501, Spectronic Camspec Ltd., Leeds, UK).

### 4.6. Strawberry Leaves Inoculation

After the fourth application (BBCH 71-77) of the test measures, randomly selected 12 strawberry leaves (visually healthy) from each treatment were artificially inoculated with *Botrytis cinerea* in vitro. The middle leaflet was selected for further experiments. The strawberry leaves were placed in a Petri dish with a sterile filter paper and 5 mL sterile distilled water. The center of the leaf was inoculated with 6 mm *B. cinerea* mycelium plugs (mycelial side downwards). *B. cinerea* prior the experiment was maintained on the potato dextrose agar (Liofilchem, Roseto degli Abruzzi, Teramo, Italy); for the experiments, a 7-day old fungus was used. The *B. cinerea* (13B_E36, host strawberry) was from the LAMMC IH Laboratory of Plant Protection isolate collection [40,46]. The Petri dish with leaves was incubated at 22 °C in the dark. The *B. cinerea* incidence was evaluated 2, 4, and 7 days after inoculation (DAI). Whole-leaf length, width (mm), and diameter of damage (mm) were measured. The *B. cinerea* damage severity was calculated as the percentage of leaf area with disease symptoms [40,46,52].

### 4.7. Statistics

The data was analyzed using the MS Excel software (Microsoft, Redmond, WA, USA). The data analyses were performed using SAS Enterprise Guide, version 7.1, developed by SAS Institute Inc. in Cary, NC, USA. The analysis of variance (ANOVA) method and Duncan’s test estimated the significance of differences between treatments at *p* < 0.05. The results are expressed as a mean ± standard deviation, with vertical bars in the figures representing the error bar of the mean.

## 5. Conclusions

This 2-year study demonstrated that applied biological protection strategies influenced strawberry growth, yield, fruit quality, antioxidant responses, and gray mold severity, with treatment efficacy strongly dependent on the growing season.

Vegetative growth was generally higher in the second year, indicating a cumulative or delayed response to applied treatments. Yield responses varied between years: thyme essential oil treatments resulted in higher total yield in 2023, whereas chemical plant protection and Biofungicide II produced the highest yields in 2024. Fruit size and weight were greater in 2024, while SSC and ascorbic acid content were higher in 2023. Bacteria consistently enhanced fruit size and weight without negatively affecting firmness or soluble solids content.

Gray mold severity caused by *Botrytis cinerea* was higher in 2023. Chemical treatments provided the strongest disease suppression, while thyme essential oil and biofungicide treatments reduced disease incidence relative to the Control, although their efficacy varied between years. The results indicate that biological products can be effectively integrated into strawberry production systems to complement chemical fungicides. Bacteria treatment with *Bacillus* spp. as a biofungicide appears suitable for improving yield stability and fruit size, while thyme essential oil may contribute to disease suppression under favorable conditions. These strategies should be applied within integrated plant protection programs to reduce chemical inputs while maintaining yield and fruit quality in the strawberry cv. Sonsation.

## Figures and Tables

**Table 1:** The effect of biological measures on the antioxidant system response of strawberry leaves and ascorbic acid content.

Treatment	DPPH mM TE g^−1^ SM	ABTS mM TE g^−1^ SM	FRAP µmol g^−1^ SM	TPC mg g^−1^ SM	Ascorbic Acid mg %
2023					
Control	864.3 ± 106.4 ab	700.1 ± 57.6 ab	1571.1 ± 81.0 ab	66.9 ± 1.8 a	51.0 ± 3.4 a
Chemical	921.9 ± 84.0 ab	754.1 ± 56.1 ab	1599.5 ± 55.6 ab	78.8 ± 4.9 abc	59.1 ± 3.2 abc
Bacteria	903.6 ± 47.8 ab	762.1 ± 33.6 ab	1535.0 ± 60.8 ab	83.0 ± 8.5 abc	59.3 ± 5.6 abc
Thyme I	939.5 ± 108.3 b	778.3 ± 52.7 b	1597.5 ± 85.2 ab	71.7 ± 1.8 abc	60.4 ± 6.1 abc
Thyme II	757.5 ± 97.9 ab	634.0 ± 62.0 ab	1466.7 ± 75.6 ab	74.1 ± 3.3 abc	58.5 ± 4.8 a
Biofungicide I	884.2 ± 48.7 ab	721.6 ± 46.3 ab	1603.1 ± 49.8 b	86.0 ± 8.9 c	75.4 ± 6.1 abc
Biofungicide II	935.5 ± 131.1 ab	687.1 ± 41.0 ab	1474.3 ± 70.6 ab	86.0 ± 5.2 abc	79.9 ± 8.2 c
2024					
Control	1132.3 ± 77.5 a	939.5 ± 56.7 a	1870.4 ± 82.2 abc	80.7 ± 6.2 abc	32.5 ± 4.3 ab
Chemical	934.7 ± 200.8 a	1121.8 ± 59.5 c	2035.2 ± 67.8 c	86.2 ± 6.6 abc	34.4 ± 4.4 ab
Bacteria	1153.7 ± 189.2 c	1025.3 ± 96.2 abc	1990.1 ± 73.0 bc	89.3 ± 2.7 c	39.3 ± 4.5 ab
Thyme I	1344.4 ± 15.2 abc	1047.1 ± 58.0 abc	1997.7 ± 28.9 bc	72.3 ± 4.6 a	37.6 ± 4.8 ab
Thyme II	1194.7 ± 76.9 abc	986.2 ± 36.9 abc	1847.3 ± 46.2 abc	72.8 ± 6.0 abc	35.9 ± 1.6 ab
Biofungicide I	1246.4 ± 27.4 abc	1088.5 ± 46.1 abc	1951.9 ± 43.7 abc	76.9 ± 6.1 abc	40.2 ± 2.5 b
Biofungicide II	1070.2 ± 39.4 a	950.9 ± 23.6 abc	1768.5 ± 45.2 a	76.6 ± 3.0 abc	39.3 ± 2.1 ab

Note. The same letter indicates no significant differences between treatments; Duncan’s test (*p* < 0.05). The results are expressed as a mean ± standard deviation.

**Table 2:** The experiment treatments, dosage, and application intervals.

Treatment	Composition	Dose	Application
Control	-	-	-
Chemical	Boscalid 267 g L^−1^ + pyraclostrobin 37 g L^−1^	1.8 kg ha^−1^	2 times (interval 7–10 days)
	Ciprodinil 375 g L^−1^ + fludioxonil 250 g L^−1^	1.0 kg ha^−1^	2 times (interval 7–10 days)
Bacteria	*B. halotolerans* Bil-LT1_1 *B. halotolerans* Bil-LT1_2 *B. velezensis* Cran-LT_1_8 *B. velezensis* Ling-NOR_4_15	~10^−6^ CFU/L	4 times (interval 7–10 days)
Thyme I	*Thymus vulgaris*	600 µL/L	4 times (interval 7–10 days)
Thyme II	*Thymus vulgaris*	600 µL/L	4 times (interval 7–10 days) + 3 times between harvests
Biofungicide I	*Bacillus subtilis* (QST 713), 13.96 g/L, 1,34%, 1.042 × 10^12^ CFU/L	8 L/ha	4 times (interval 7–10 days) + 3 times between harvests
Biofungicide II	*Clonostachys rosea* J1446 900 g/kg (90–96%)	220 g/ha	4 times (interval 7–10 days)

**Table 3:** The environmental conditions—air temperature and humidity.

Environmental Conditions		2023			2024		
		April	May	June	April	May	June
Air temperature, °C	Min	−6.65	−4.13	2.4	−1.18	−0.99	6.20
	Max	49.27	54.53	49.96	44.71	44.71	51.39
	Average	13.59	20.33	24.42	18.90	19.53	24.43
Air humidity, %	Min	13.16	9.40	10.7	14.20	14.20	15.98
	Max	100.00	97.21	98.4	97.57	97.56	96.71
	Average	68.91	53.52	57.16	58.90	58.32	60.08

## Data Availability

The data presented in this study are available upon request from the corresponding author.

## Abbreviations

The following abbreviations are used in this manuscript:

DPI	days post inoculation
Control	untreated, control
Chemical	treatment were used chemical fungicides, Boscalid 267 g L^−1^ + pyraclostrobin 37 g L^−1^ and Ciprodinil 375 g L^−1^ + fludioxonil 250 g L^−1^
Bacteria	used four bacteria mixture, *B. halotolerans* Bil-LT1_1, *B. halotolerans* Bil-LT1_2, *B. velezensis* Cran-LT_1_8, and *B. velezensis* Ling-NOR_4_1
Thyme I	*Thymus vulgaris* essential oil, used four times
Thyme I	*Thymus vulgaris* essential oil, used four times, plus three times between harvesting
Biofungicide I	used biological fungicide *Bacillus subtilis* (QST 713), used four times, plus three times between harvestings
Biofungicide II	used biological fungicide *Clonostachys rosea* J1446, used four times
EO	essential oil
TPC	total phenolic compounds
SCC	soluble solids content
ABTS	2,2′-azino-bis (3-ethylbenzothiazoline-6-sulphonic acid
TE	Trolox equivalents
SM	dry mass
DPPH	2-diphenyl-1-picrylhydrazyl
FRAP	Fe^2+^ (Ferric) reducing antioxidant power
